# Enhance Portable Radiograph for Fast and High Accurate COVID-19 Monitoring

**DOI:** 10.3390/diagnostics11061080

**Published:** 2021-06-12

**Authors:** Ngan Le, James Sorensen, Toan Bui, Arabinda Choudhary, Khoa Luu, Hien Nguyen

**Affiliations:** 1 Department of CSCE, University of Arkansas, Fayetteville, AR 72701, USA; khoaluu@uark.edu; 2 Department of Radiologist, University of Arkansas for Medical Sciences UAMS, Little Rock, AR 72205, USA; JISorensen@uams.edu (J.S.); AChoudhary@uams.edu (A.C.); 3 Vin-AI Research, Hanoi 100000, Vietnam; v.toanbd1@vinai.io; 4 Department of CSCE, University of Houston, Houston, TX 77204, USA; hvnguy35@central.uh.edu

**Keywords:** COVID-19, CXR, chest X-ray, portable CXR, conventional CXR, alignment, enhancement, deep learning, artificial intelligence

## Abstract

This work aimed to assist physicians by improving their speed and diagnostic accuracy when interpreting portable CXRs as well as monitoring the treatment process to see whether a patient is improving or deteriorating with treatment. These objectives are in especially high demand in the setting of the ongoing COVID-19 pandemic. With the recent progress in the development of artificial intelligence (AI), we introduce new deep learning frameworks to align and enhance the quality of portable CXRs to be more consistent, and to more closely match higher quality conventional CXRs. These enhanced portable CXRs can then help the doctors provide faster and more accurate diagnosis and treatment planning. The contributions of this work are four-fold. Firstly, a new database collection of subject-pair radiographs is introduced. For each subject, we collected a pair of samples from both portable and conventional machines. Secondly, a new deep learning approach is presented to align the subject-pairs dataset to obtain a pixel-pairs dataset. Thirdly, a new PairFlow approach is presented, an end-to-end *invertible* transfer deep learning method, to enhance the degraded quality of portable CXRs. Finally, the performance of the proposed system is evaluated by UAMS doctors in terms of both image quality and topological properties. This work was undertaken in collaboration with the Department of Radiology at the University of Arkansas for Medical Sciences (UAMS) to enhance portable/mobile COVID-19 CXRs, to improve the speed and accuracy of portable CXR images and aid in urgent COVID-19 diagnosis, monitoring and treatment.

## 1. Introduction

Chest radiography, also known as chest X-ray or CXR, is among the most common forms of medical imaging. Typically, portable CXR is performed on acutely ill patients whose conditions are too critical or unstable to be transported to a radiology facility for a conventional chest X-ray. However, in the setting of the COVID-19 pandemic, the American College of Radiology guidelines now list portable CXRs as the preferred imaging modality for the investigation of novel coronavirus patients [1,2]. This involves a portable X-ray machine being brought into the patient’s room, and subsequently decontaminated, which reduces the risk of disease transmission compared to having the patient come to the radiology department for conventional CXR. The existence of portable CXR systems aims to acquire images within an isolation room, thus significantly reducing the risk of COVID-19 transmission during transport to fixed systems such as CT scanners, as well as within the rooms housing the fixed imaging systems [3]. Furthermore, some have found portable CXRs to be highly valuable for critically ill COVID-19 patients [4]. Portable CXR is used to monitor patients in intensive care units (ICUs) which are more than 5% of the total known cases of COVID-19. The potential drawbacks and difficulties of portable CXRs have been recognized and discussed elsewhere [5,6].

**Figure 1:**
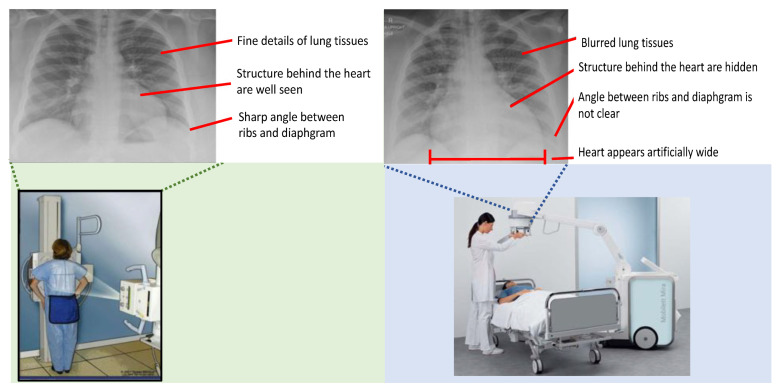
Comparison of conventional (left) vs. portable (right) CXR acquisition. The conventional CXR (left) is shown with high-quality details in lung tissue, well-defined structures behind the heart, and a sharp angle between the ribs and diaphragm. The portable CXR (right) shows degraded features, with blurred lung tissues, obscured structures behind the heart, a blurred angle between ribs and diaphragm, and an artificially wide appearance of the heart.

Portable CXRs, as shown in Figure 1 right, have reduced diagnostic accuracy when compared to conventional radiographs, as shown in Figure 1 left, with inconsistent patient positioning, suboptimal anatomic alignment, and unpredictable beam penetration, all reducing image quality [7]. Conventional CXR studies are ideally performed with the patient in standing position, with the X-ray source and a long distance behind the patient (posterior-to-anterior or PA projection), while portable radiographs are obtained with the patient sitting or lying and with the X-ray source close in front of them (anterior-to-posterior or AP projection). This commonly results in artifacts such as geometric magnification of the heart. Moreover, as described in [8,9], the degradation of image quality occurs most frequently in chest imaging as a result of improper collimation, a problem to which portable radiographs are particularly prone. This results in a large percentage of the photons entering the chest engaging in Compton interactions and resulting in forward-scatter, causing a noise-laden, low-frequency background signal that creates a visible haze. Thus, portable radiographs typically demonstrate reduced contrast and spatial resolution. This creates the potential for obscured vasculature, infiltrates and other pathologies [8,10]. 

**Figure 2:**
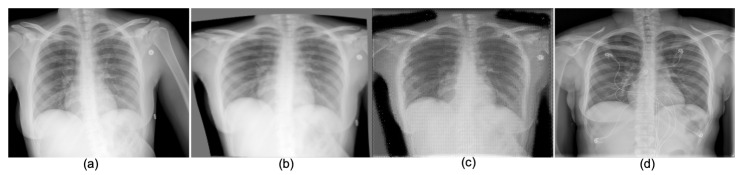
An example of an enhanced quality portable CXR, of the kind used for COVID-19 patients: (**a**) original portable CXR; (**b**) aligned CXR from (**c**); (**c**) quality-enhanced CXR from (**b**); and (**d**) a reference of high-quality CXR captured from a conventional machine.

An illustration comparing conventional CXRs and portable CXRs is given in Figure 1 and Figure 2. As shown in Figure 1 left and Figure 2d, the radiographs from the conventional machine are shown in high quality with fine details in lung tissues, well-defined structures behind the heart, and a sharp angle between the ribs and diaphragm. The portable radiographs in Figure 1 right and Figure 2a are shown in **lower quality with blurred lung tissues, structures obscured behind the heart**, and **blurred angle between the ribs and diaphragm**. Furthermore, patient positioning also affects the image quality and diagnosis results. As shown in Figure 1 right, the **heart appears artificially wider** with anterior-to-posterior beam orientation used in portable imaging, when compared to conventional posterior-to-anterior projection, and is worsened by the closed proximity of the source in portable imaging. Enhancing portable radiographs quality is a desirable task not only used for the imaging of COVID-19 patients, which is expected to continue increasing in the coming months, but is also applicable to other patients in an ICU, nursing home, corrections facility, or another location where portable radiography is frequently used [7].

Deep neural networks (DNNs) are a recent development in Artificial Intelligence (AI), and have set the state-of-the-art performance in many tasks in computer vision and biomedical imaging. In this work, we intended to develop a new **DNN-based domain translation network**, named **PairFlow** as a generative model to exploit and learn the images from conventional radiograph machines (target domain), and use this to align and enhance the images from portable radiograph machines (source domain). The goal of our proposed DNN-based domain translation network is to learn a conditional mapping function, which is able to transfer the knowledge, i.e., presented by image quality, from a good quality domain to a degraded quality domain. In addition, our proposed DNN-based approach also contains a **alignment model** which aims at transforming portable radiograph alignment to conventional radiograph alignment to prevent topological errors. Both components, namely the alignment network and the PairFlow knowledge translation network, are trained in deep learning frameworks. Given a degraded portable CXR (Figure 2a), our proposed network first performs an alignment to obtain the aligned CXR through the first component (Figure 2b). Then, the CXR quality is enhanced by the second enhancement component, i.e., the knowledge translation network (Figure 2c), while (Figure 2d) is considered a preferred high-quality CXR from the conventional machine.

**Contributions of this work:** In this paper, we developed a novel deep learning approach to align and enhance the quality of portable CXRs to an appearance consistent with conventional CXRs. Our work aimed to help physicians to improve their speed and diagnostic accuracy when reading portable CXRs, which are in especially **high demand** in the current context of COVID-19 pandemic, in which the number of imaging studies can dramatically increase in a matter of days. The contributions of this work are four-fold:

- Firstly, we introduce a novel **database collection of subject-pairs radiographs**. For each subject, we collect a pair of samples which are from both portable machines (source domain) and conventional machines (target domain).
- Secondly, we introduce a new deep learning-based approach to **align a subject-pairs dataset to obtain pixel-pairs dataset**. In order to learn the knowledge correlation between two different domains, it is important to have a pixel-wise pair dataset. Thus, alignment is an important step that helps to perform knowledge transferring from the source domain to target domain.
- Thirdly, we propose a new **PairFlow** approach, an end-to-end *invertible* transfer of a deep learning method, to enhance the degraded CXRs from the portable machine. High-quality knowledge is then transferred to a degraded domain to increase the portable CXRs quality.
- Finally, we evaluate the system performance at both **image-quality enhancement and topological properties**.

## 2. Related Works and Background

In medical imaging and computer vision, the task of producing a high-quality image from a low-quality image is called an image-to-image translation. Image-to-image translation has recently gained attention in the medical imaging community, where the task is to estimate the corresponding image in the target domain from a given source domain image of the same subject. Generally, image-to-image translation methods can be divided into two categories including: generative adversarial networks (GANs) and flow-based generative networks, as summarized in the following subsections.

### 2.1. Generative Adversarial Networks

Generative adversarial networks (GANs) are a class of latent variable generative models that clearly identify the generator as *deterministic mapping*. The deterministic mapping represents an image as a point in the latent space without regarding its feature ambiguity. Several different GAN-based models have been used to explore image-to-image translation in a literature study [11,12,13]. For example, Zhu et al. [13] proposed a cycleGAN method for mapping between unpaired domains by using cycle-consistency dependence to constrain the optimal solutions provided by the generative network. Chen et al. [11] proposed a 3D cycleGAN network to learn the mapping between CT and MRI. The drawback of 3D cycleGAN is its high memory consumption and loss of global information due to working on small patch sizes.

### 2.2. Flow-Based Generative Networks

Flow-based generative networks are a class of latent variable generative models that clearly identify the generator as an *invertible mapping*. The invertible mapping provides a distributional estimation of features in the latent space. Recently, many efforts making use of flow-based generative networks have been proposed to transfer between two unpaired data [14,15,16,17,18]. For example, Grover et al. [15] introduced a flow-to-flow (flow2flow) network for unpaired image-to-image translation. Sun et al. [18] introduced a conditional dual flow-based invertible network to transfer between positron emission tomography (PET) imaging and magnetic resonance imaging (MRI) images. By using invertible properties, the flow-based methods can ensure exact cycle consistency in translation from a source domain to the target and returning to the source domain without any further loss functions.

### 2.3. Comparison between GANs (cycleGAN) and Flow-Based Generative Networks

**Figure 3:**
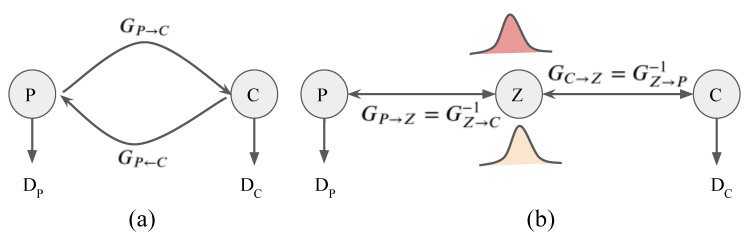
A comparison between (**a**) cycleGAN and (**b**) flow-based generative model. Double-headed arrows denotes invertible mapping.

Let ${\left\{c_{i}\right\}}_{i=1}^{N}$ and ${\left\{d_{i}\right\}}_{i=1}^{M}$ be unpaired data samples for two domains, i.e., the source domain *P* (CXRs from portable machines) and the target domain *C* (CXRs from conventional machines), respectively. Denote *D* and *G* as a discriminator network and a generator network, respectively. The cycleGAN model [13] solves unpaired image-to-image translation between these two domains by estimating two independent mapping functions $G_{P\to C}:P\to C$ and $G_{C\to P}:C\to P$. The two mapping functions $G_{P\to C}$ and $G_{C\to P}$ performed by neural networks are trained to fool the discriminators $D_{P}$ and $D_{C}$, respectively. The discriminator $D_{P}$, and $D_{C}$ encourage the transferred images and the real images to be similar, as given in Figure 3a.

Flow-based generative models [19,20,21] are a class of latent variable generative models that clearly identify the generator as an invertible mapping h:Z→P between a set of latent variables *Z* and a set of observed variables *P*. Let $p_{P}$ and $p_{Z}$ indicate the marginal densities given by the model over *P* and *Z*, respectively. Using the change-of-variables formula, these marginal densities are defined as

(1) $$p_{P}\left(x\right)=p_{Z}\left(z\right){\left|\det\frac{\partial h^{-1}}{\partial P}\right|}_{P=p}$$

where $z=h^{-1}\left(x\right)$ because of the invertibility constraints. In particular, we use a multivariate Gaussian distribution $p_{Z}\left(z\right)=N\left(\mu ,\;0,\;I\right)$. Unlike in adversarial training, flow models trained with maximum likelihood estimation (MLE) explicitly require a prior $p_{Z}\left(z\right)$ with a tractable density to evaluate model likelihoods using the change-of-variables formula (Equation (1)).

Based on a flow-based method [14], Grover et al. [15] proposed a flow2flow method for unpaired image-to-image translation. In the method, the mapping between two domains from X→Y can be represented through a shared feature space of latent variables *Z* by the composition of two invertible mappings [15]:

(2) $$G_{P\to C}=G_{Z\to C}G_{P\to Z},\;\mathrm{and}\;G_{C\to P}=G_{Z\to P}G_{C\to Z}$$

where $G_{P\to Z}=G_{Z\to P}^{-1}$ and $G_{C\to Z}=G_{Z\to C}^{-1}$, as given in Figure 3b. Due to the fact that the composition of invertible mappings is invertible, both $G_{P\to C}$ and $G_{C\to P}$ are invertible [15]. On the other hand, we can obtain $G_{P\to C}^{-1}=G_{C\to P}$.

Figure 3 illustrates the difference between cycleGAN and flow-based methods. Unlike cycleGAN, the flow-based method is the full invertible architecture that guarantees the cycle-consistency translations between two unpaired domains. Our proposed PairFlow network is an improvement of flow-based deep learning method.

More details about the comparison between GAN and flow-based generative model networks are reported in [22].

### 2.4. Deep Learning for COVID-19 on CXR

At present, CXR is extensively used for the detection of the COVID-19 cases compared to the CT image as it takes longer for imaging, and CT scanners are not available in many underdeveloped countries [23]. In last couple of months, a large number of researchers have investigated and analyzed CXR images using deep learning algorithms to detect and diagnose COVID-19. In this section, we discuss some recent advanced deep learning techniques for both COVID-19 detection and COVID-19 classification. COVID-19 detection using CXR has been well studied in [24,25,26,27,28,29]. Furthermore, the classification of COVID-19 from CXR has been well studied in [30,31,32,33]. Most of these methods use off-the-shelf networks, including VGG-16 or VGG-19 [34,35], ResNet [36] variants such as ResNet-18, ResNet-50, ResNet-101, ResNet-151 [30,37,38], Inception [33], EfficientNet [39,40], DenseNet [41,42]. While other networks show promising results, ResNet and DenseNet obtain better performance than the others, with accuracies ranging from 88% to 99%. There are three common classes considered, i.e., COVID-19, non-COVID-19 pneumonia and normal in such research. Most of the reported work in the literature has used CXR images to detect and diagnose COVID-19, and this highlights the importance of CXR image analysis as a positive tool for doctors and specialists.

There have been important recent efforts to push for open access and open source solutions for CXR-driven COVID-19 case detection and classification [29,43,44,45,46]. Among these datasets, COVID-Net [29], which is considered as one of the largest CXR datasets for the pandemic study, leverages the human–machine collaborative design strategy to conduct the dataset.

Recent guidelines [1,2] of the North American Radiology Scientific Expert Panel have assessed that portable CXR has to be considered as the main imaging approach in evaluating COVID-19 patients. Using portable CXR does not only reduce radiation for patients, but also prevents the need to transport them. Furthermore, portable CXR is also used to monitor patients in intensive care units (ICUs) which are more than 5% of the total known cases of COVID-19. However, the image degradation problem in the portable CXR imaging compared to conventional CXR imaging has not been studied well in the literature review. In this work, we tackle this problem by proposing a FairFlow network architecture, a flow-based network for enhancing portable CXR images.

## 3. Our Proposed Method

In order to help doctors provide fast and highly accurate COVID-19-related diagnostic information from portable CXRs, as well as monitor the treatment process, this work aims to enhance the quality of portable CXRs to be approximately equal to the quality of the conventional CXRs. Therefore, this section will include two main tasks, i.e., radiograph alignment and radiograph quality enhancement via two new deep learning networks. In our proposed network, we used the following notations:

- *C*: target domain—CXR from conventional machines;
- $I_{C}$: each CXR in the target domain *C*;
- *P*: source domain—CXR from portable machines;
- $I_{P}$: each CXR in the source domain.

### 3.1. Portable Radiograph Alignment

**Figure 4:**
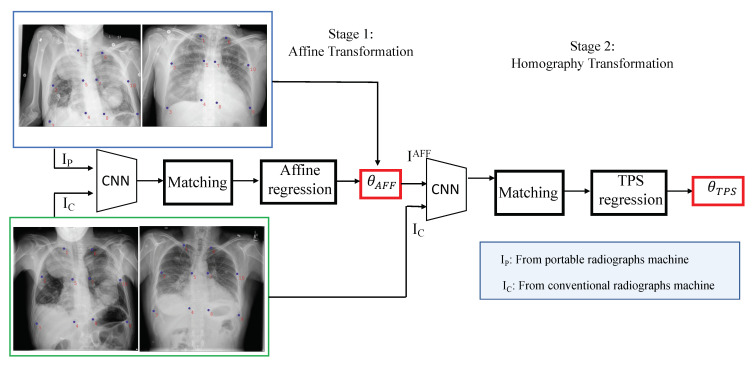
The flowchart of training portable radiograph alignment. The input is a pair-subject dataset and the model output is affine transformation (six degrees-of-freedom) $\theta_{AFF}$ and homography transformation $\theta_{TPS}$.

Figure 4 illustrates the presented framework of portable radiograph alignment. We aimed to align the portable image ($I_{P}$) to the conventional diagnostic ($I_{C}$) with a large transformation; hence, it is difficult to obtain a good alignment with a single-stage transformation. In this paper, we addressed the problem using two-stage transformations: affine and thin-plate spline (TPS) transformations [47].

We obtained the affine transformation between two images by estimating six degree-of-freedom linear transformation $\phi_{AFF}=\left[\alpha_{1},\alpha_{2},\alpha_{3},\alpha_{4},t_{1},t_{2}\right]$ from extracted features by the Resnet network [36]. The affine transformation is capable of modeling translation, rotation, non-isotropic scaling and shear between two images, $I_{P}$ and $I_{C}$, and can be formulated as follows:

(3) $$I^{AFF}=\left[\begin{array}{cc} \alpha_{1} & \alpha_{2}\\ \alpha_{3} & \alpha_{4} \end{array}\right]I_{P}+\left[\begin{array}{c} t_{1}\\ t_{2} \end{array}\right]$$

**Figure 5:**
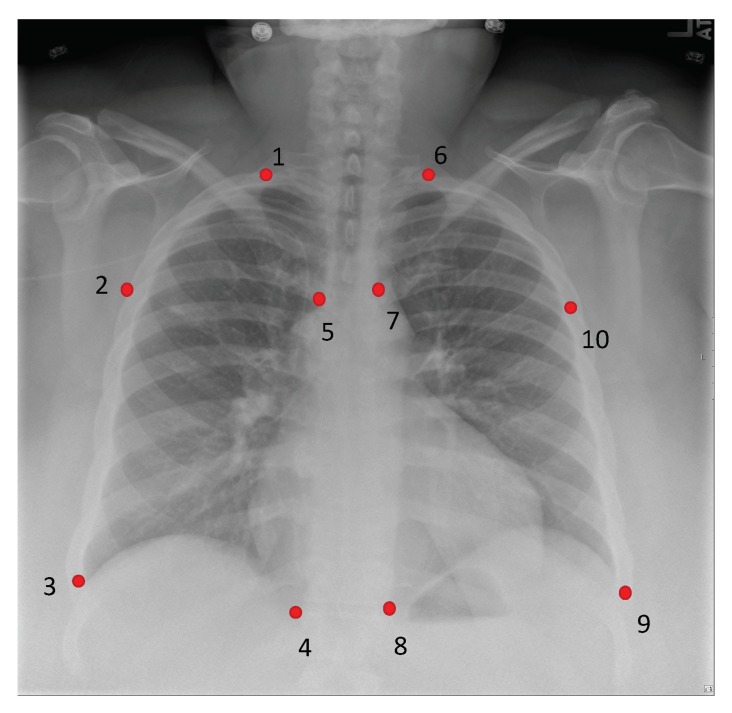
Our proposed CXR landmarking scheme. There are 10 keypoints defined in our landmarking scheme.

The aligned image $I^{AFF}$ then passed through the second thin-plate spline (TPS) transformation to obtain $I^{AFF+TPS}$. It performs a smooth 2-D interpolation of a given set of *k* (k=10 in this work) of landmark points $P_{M}=\left[P_{M1},\ldots ,P_{Mk}\right]$ in the portable image and $C_{M}=\left[C_{M1},\ldots ,C_{Mk}\right]$ in the conventional diagnostic. Our landmarking scheme is illustrated in Figure 5 the TPS can be parametrized by a 18 dimensional vector of the aligned source portable image $I^{AFF}$ by $\phi_{TPS}=\left[x_{P_{M1}},y_{P_{M1}},\ldots ,x_{P_{Mk}},y_{P_{Mk}}\right]$.

### 3.2. Portable Radiograph Quality Enhancement

**Figure 6:**
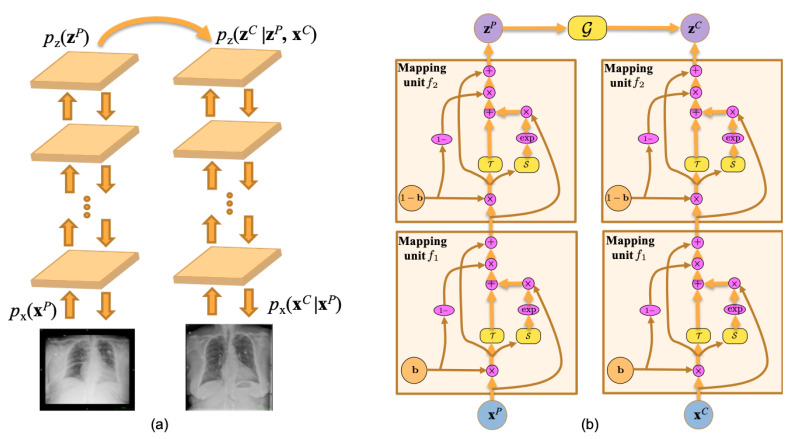
Our proposed PairFlow deep network for image enhancement between the source domain (P) and target domain (C): (**a**) invertible CNN-based PairFlow Network for portable radiograph enhancement; and (**b**) a mapping unit *f* whose transformations S and T are represented with a one-residual-block CNN network.

Let $I\subset R^{C}$ be the radiograph domain and $\{x^{P},x^{C}\}\in I$ be observed variables encoding in *P* and *C*, respectively, In order to embed the flow transformation between the conventional domain (*C*) and portable domain (*P*), a bijection mapping function is defined to map from the radiograph space I to a latent space Z and then model the relationship between these latent variables. Mathematically, let F:I→Z denote a bijection from an given radiograph variable x to its corresponding latent variable z and G:Z→Z be an radiograph transformation function modeling the radiograph relationships between variables in the latent spaces. As shown in Figure 6a, our PairFlow network consists of three main components: Two bijection functions $F_{1},F_{2}:X\to Z$ present the mapping from observed radiograph variables $x^{C},x^{P}$ to their latent variables $z^{C},z^{P}$, respectively; and a radiograph transformation function G:Z→Z between variables in the latent spaces. The relationships between variables can be defined as follows:

(4) $$\begin{array}{cc} z^{C} & =F_{1}\left(x^{C};\theta_{1}\right)\\ z^{P} & =H(z^{C},x^{P};\theta_{2},\theta_{3})\\ & =G\left(z^{C};\theta_{3}\right)+F_{2}\left(x^{P};\theta_{2}\right) \end{array}$$

In Equation (4), $F_{1},F_{2}$ denote the mappings of $x^{C}$ and $x^{P}$ to their latent variables of radiographs, respectively. H is the summation of $G(z^{C};\theta_{3})$ and $F_{2}\left(x^{P};\theta_{2}\right)$. Given a conventional diagnostic CXR $x^{C}$, the probability density function can be formulated as in Equation (5):

(5) $$\begin{array}{c} \begin{array}{cc} p_{X^{P}}\left(x^{P}|x^{C};\theta \right) & =p_{X^{P}}\left(x^{P}|z^{C};\theta \right)\\ & =p_{Z^{P}}\left(z^{P}|z^{C};\theta \right)\left|\frac{\partial H(z^{C},\;x^{P};\theta_{2},\theta_{3})}{\partial x^{P}}\right|\\ & =p_{Z^{P}}\left(z^{P}|z^{C};\theta \right)\left|\frac{\partial F_{2}\left(x^{P};\theta_{2}\right)}{\partial x^{P}}\right| \end{array} \end{array}$$

In Equation (5), $p_{X^{P}}\left(x^{P}|x^{C};\theta \right)$ and $p_{Z^{P}}\left(z^{P}|z^{C};\theta \right)$ denote the distribution of $x^{P}$ conditional on $x^{C}$ and the distribution of $z^{P}$ conditional on $z^{C}$, respectively. The second equality in Equation (5) can be computed using the change of variable formula, and $\frac{\partial F_{2}\left(x^{P};\theta_{2}\right)}{\partial x^{P}}$ is the Jacobian. In this formulation, the assigned task can be accomplished by computing the density of its corresponding latent point $z^{P}$ given $z^{C}$ associated with the Jacobian determinant $\left|\frac{\partial F_{2}\left(x^{P};\theta_{2}\right)}{\partial x^{P}}\right|$.

Such a bijection function can produce a large Jacobian matrix, thus its computation is extremely expensive. In order to achieve the tractable property at a lower computational cost, we construct F as a composition of tractable mapping units $f_{F}$. Each mapping unit is built from multiple convolution layers that will form a deep convolutional neural network of the bijection function F. The details of the bijection function are introduced in the following section.

#### 3.2.1. Mapping Function via ResNet Layers

In general, F is presented as a composition tractable mapping unit *f* where each unit can be represented as a combination of several convolutional layers. Then, the bijection function F can be formulated as a deep convolutional neural network (CNN).

**ResNet-based Mapping Unit:** In order to make the model tractable and computationally efficient, a bijection unit *f* is defined as follows. Given an input x, a unit f:x→y defines a mapping between x and an intermediate latent state y as in Equation (6):

(6) $$\begin{array}{c} y=x^{\prime }+\left(1-b\right)⊙\left[x⊙\exp\left(S\left(x^{\prime }\right)\right)+T\left(x^{\prime }\right)\right] \end{array}$$

In Equation (6), $x^{\prime }=b⊙x$; b=[1,⋯,1,0,⋯,0] is a binary mask where the first *d* elements of b is set to one and the rest is zero; S and T represent the scale and the translation functions, respectively; and ⊙ denotes the Hadamard product. The Jacobian of this transformation unit can be computed as

(7) $$\begin{array}{cc} \frac{\partial f}{\partial x} & =\left[\begin{array}{cc} \frac{\partial y_{1:c}}{\partial x_{1:c}} & \frac{\partial y_{1:c}}{\partial x_{c+1:C}}\\ \frac{\partial y_{d+1:C}}{\partial x_{1:c}} & \frac{\partial y_{c+1:C}}{\partial x_{c+1:C}} \end{array}\right]\\ \; & =\left[\begin{array}{cc} I_{c} & 0\\ \frac{\partial y_{c+1:C}}{\partial x_{1:c}} & \mathrm{diag}\left(\exp(S\left(x_{1:c}\right))\right) \end{array}\right] \end{array}$$

where $\mathrm{diag}\left(\exp(S\left(x_{1:c}\right))\right)$ is the diagonal matrix such that $\exp(S\left(x_{1:c}\right))$ is their diagonal elements. The above equation introduces two important features for the mapping unit *f*. The form of Jacobian matrix $\frac{\partial f}{\partial x}$ can be well defined as triangular, and the determinant of this matrix can be computed shortly. The tractable feature is also guaranteed for *f*. The Jacobian of two functions S and T are also not required in the computation of $\left|\frac{\partial f}{\partial x}\right|$. Thus, S and T can be formulated with any non-linear function. In this work, the functions S and T are formulated as a composition of residual networks in ResNet. This ResNet-style framework therefore allows high-level radiograph features to be efficiently extracted in the mapping, as shown in Figure 6b. On the other hand, apart from other traditional deep learning frameworks, the inverse function $f^{-1}:y\to x$ in this work can be simply derived as follows:

(8) $$\begin{array}{cc} x= & y^{\prime }+\left(1-b\right)⊙\left[\left(y-T\left(y^{\prime }\right)\right)⊙\exp\left(-S\left(y^{\prime }\right)\right)\right] \end{array}$$

where $y^{\prime }=b⊙y$.

**Bijective Mapping Function:** The bijective mapping F can be derived as a combination of the sequence of mapping units $\left\{f_{1},f_{2},\cdots ,f_{n}\right\}$, i.e., $F=f_{1}\circ f_{2}\circ \cdots \circ f_{n}$. In order to derive the Jacobian of F, its units are simply computed with the guarantee of tractable property:

(9) $$\begin{array}{c} \frac{\partial F}{\partial x}=\frac{\partial f_{1}}{\partial x}\cdot \frac{\partial f_{2}}{\partial f_{1}}\cdots \frac{\partial f_{n}}{\partial f_{n-1}} \end{array}$$

In this framework, each mapping unit is set up as a composition of CNN layers. Therefore, the bijection F as shown in Figure 6a can be formulated as a CNN network to map the observed radiograph variable x∈I to a latent variable z∈Z.

#### 3.2.2. The Radiograph Enhancement Embedding

In Section 3.2.1, the invertible mapping function F is presented between a radiograph data distribution $p_{X}$ and a latent distribution $p_{Z}$. In this subsection, $p_{Z}$ is presented as a *Gaussian distribution* to model the variations in radiographs, but our proposed model is able to work well with any type of prior distributions. In addition, we further assume that the joint distribution of $z^{C}$ and $z^{P}$ embedding the relationship between the variables is also a Gaussian. The transformation $G:z^{P}\to z^{C}$ can be formulated as follows:

(10) $$G\left(z^{P};\theta_{3}\right)=Wz^{P}+b_{G}$$

where $\theta_{3}=\left\{W,b_{G}\right\}$ is the transform parameters representing connecting weights of latent-to-latent interactions and the bias.

#### 3.2.3. Enhancement Model Learning

The parameters $\theta =\{\theta_{1},\theta_{2},\theta_{3}\}$ of the model are optimized to maximize the log-likelihood as follows:

$$\begin{array}{c} \begin{array}{cc} & \log p_{X^{P}}\left(x^{P}|x^{C};\theta \right)=\log p_{Z^{P}}\left(z^{P}|z^{C},\theta \right)+\log\left|\frac{\partial F_{2}\left(x^{P};\theta_{2}\right)}{\partial x^{P}}\right|\\ = & \log p_{Z^{P},Z^{C}}\left(z^{P},z^{C};\theta \right)-\log p_{Z^{C}}\left(z^{C};\theta_{1}\right)+\log\left|\frac{\partial F_{2}\left(x^{P};\theta_{2}\right)}{\partial x^{P}}\right| \end{array} \end{array}$$

where the first two terms denote the two density functions. The third term, i.e., the determinant, can be computed efficiently. The optimal parameter values in this framework can be solved using the stochastic gradient descent (SGD) algorithm.

Although the proposed PairFlow shares some similar features with RBM and its family such as TRBM, i.e., a probabilistic graphical model with log-likelihood optimization, the log-likelihood estimation of PairFlow is tractable while that in RBM is intractable and requires some approximations during the training process. Compared to other methods, PairFlow also shows its advantages as a high-quality synthesized radiograph avoiding the $\ell_{2}$ reconstruction error which occurs with the *Variational Autoencoder* and efficient training process, i.e., avoiding finding a balance between the generator and discriminator as in GAN-based algorithms.

## 4. Experimental Results

### 4.1. Database

We collected a subject-pair X-ray dataset from 123 patients with both negative and positive tests for COVID-19, each of whom had portable and conventional images acquired either (i) within 24 h of each other and reported by a board-certified radiologist as not having changed in that span; or (ii) within 12 months of each other and both having been read as normal. Only AP and PA projection views were included. Portable images were acquired using Philips MobileDiagnost series at 90 keV, and conventional images were acquired on a Philips SRO 33100 ROT 360 at 125 keV. The images used in our experiments have the resolution of 0.148mm×0.148mm and size of 2846×2198 pixels. We randomly divided the dataset into 40 subjects for training and five subjects for validation.

### 4.2. Implementation Details

Our proposed network was implemented using the Pytorch framework and trained on a 48GB GPU machine. The input image was resized to 512×512 and normalized to [−1,1]. The Adam optimizer with a batch size of two was used to train the network. The initialization learning rate was set to 0.0002 and was decreased ten times every 20 epochs. We trained the model for 60 epochs. We set the scale number to 2, and the number of blocks was 4.

### 4.3. Results and Discussion

In this section, we provide the experimental results together with the discussions. Our proposed network contains two components corresponding to the alignment network and the enhancement network. The results of each component and the results from the entire system are discussed as follows:

#### 4.3.1. CXR Alignment Network

**Figure 7:**
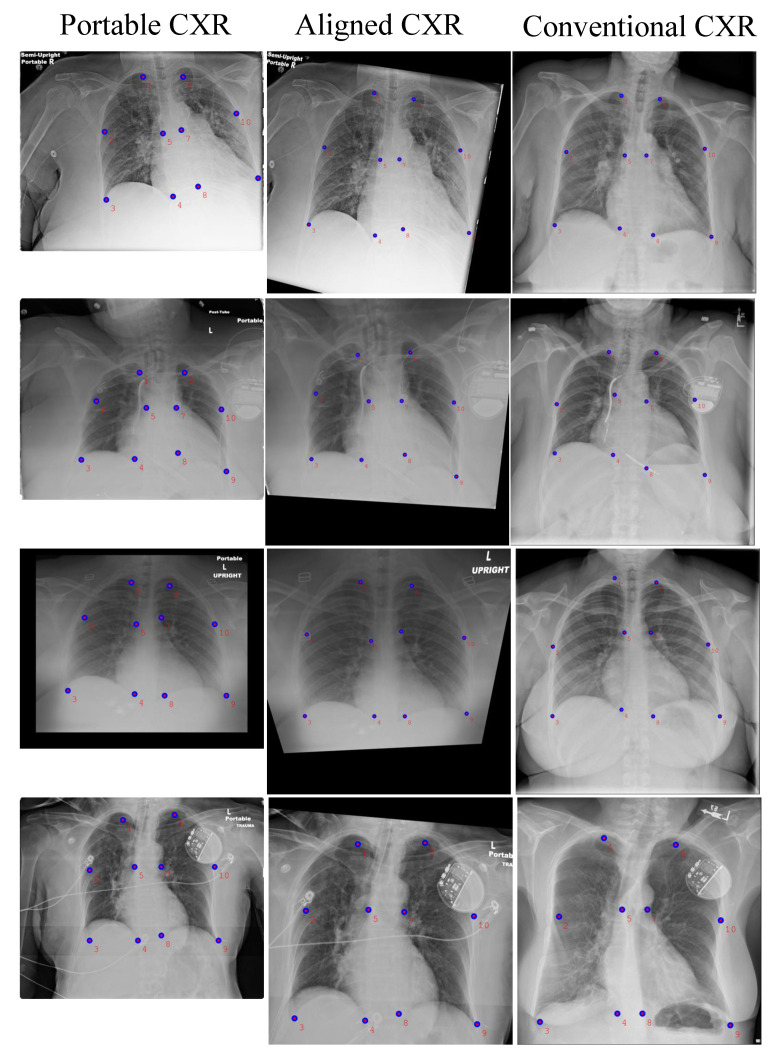
Some illustrations of our aligning results. From left to right—the first column: portable radiographs; the second column: aligned radiographs by our model; and the third column: conventional radiographs which are used for comparison.

**Figure 8:**
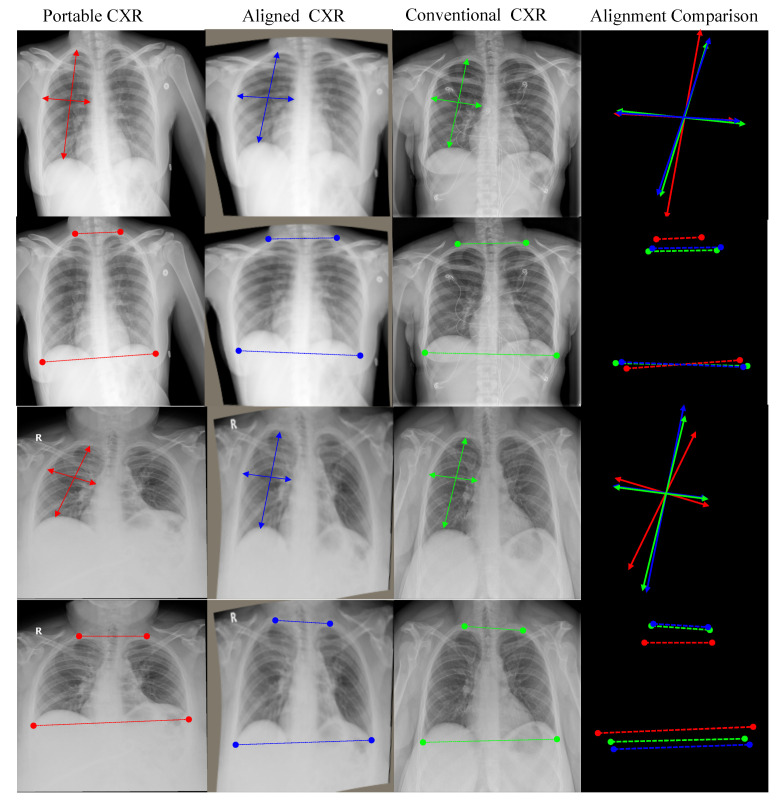
Some illustrations of our aligning results. From left to right—the first column: portable radiographs; the second column: aligned radiographs by our model; the third column: conventional radiographs which are used to compare; the fourth column: comparison between the original portable CXR, the conventional CXR and our aligned results in terms of topological information. From top to bottom—the first and second rows are the aligning results of the first subject and the third and fourth rows are the aligning results of the second subject. The first and third rows are for local topological comparison and the second and fourth rows are for global topological comparison.

As shown in Figure 1, in contrast to the conventional imaging acquisition process where the patient is standing up, portable CXR is obtained when the patient is lying down. One of the big issues is the topological change between portable CXR and conventional CXR while fluids diffuse themselves across the surface of the lung. Our proposed alignment network aims at aligning portable CXRs. Some empirical results are given in Figure 7 and Figure 8. Figure 7 shows landmark points detected on both portable CXR and aligned CXR in the first and second columns where the last column shows the landmark points on conventional CXR used as a reference. The aligned CXR together with its landmark points were obtained by applying our alignment process (Section 3.1). In Figure 8, there are two subjects. For each subject, we make two comparisons between the original portable CXR (the first column), the conventional CXR (the third column) and our aligned results (the second column) in terms of topological properties. The comparison on local topological information is given in the first row (subject 1) and the third row (subject 2) whereas the comparison of global topological information is given in the second row (subject 1) and the fourth row (subject 2). The comparison of local topological property is considered as the difference in individual lung (right lung is used to demonstrated in Figure 8) whereas the comparison of global topological property is measured as the difference in both lungs (the most top margin and the most bottom margin are used to demonstrated in Figure 8). Quantitative results of CXR alignment is evaluated using the mean absolute error (MAE) metric. The MAE between two images $I_{1}$ and $I_{2}$ is defined as

(11) $$MAE\left(I_{1},I_{2}\right)=\frac{1}{k}\sum_{i=0}^{k}\left|\right|I_{1}^{Mi}-I_{2}^{Mi}{\left|\right|}_{l1}$$

where $I_{1}^{Mi}$ and $I_{2}^{Mi}$ are the landmark points on images $I_{1}$ and $I_{2}$, respectively. *k* is the number of landmark points and k=10 in our work. In Table 1, the first column provides the MAE values between the original portable CXR and conventional CXR whereas the second column provides the MAE values between the aligned portable CXR and conventional CXR. The MAE score is both locally evaluated for each individual lung and globally evaluated for both lungs. MAE illustrates the mis-alignment between the two sets of landmark points, thus, the smaller value of $MAE(I_{1},I_{2})$ shows that landmark points on $I_{1}$ are more similar to the landmark points on $I_{1}$. The MAE score in Table 1 demonstrates that our alignment network provides an aligned portable CXR, whose topological properties are quite close to those of conventional CXR.

#### 4.3.2. Portable Radiographs Quality Enhancement

**Figure 9:**
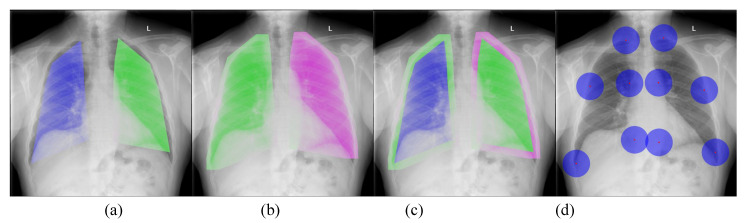
Visualization of region of interest in image quality evaluation. From left to right (**a**): inner lung region ($R_{i}^{l}$ and $R_{i}^{r}$); (**b**): outer lung region($R_{o}^{l}$ and $R_{o}^{r}$); (**c**) overlapping between inner and outer lung regions; and (**d**): area around landmark points ($R_{p}^{i}$, i∈[1,..,10]).

In medical images, especially in our present radiograph work, degradation is irregular and does not follow any specific distribution, thus a benchmark enhancement technique of the entire image may not be appropriate. In this work, the enhanced image quality was evaluated within some particular regions of interest (RoI) as given in Figure 9. The peak signal-to-noise ratio (PSNR) metric defined in Equation (12) and the structural similarity index [48] defined in Equation (13) are two metrics used to quantitatively benchmark our degradation enhancement performance. PSNR and SSIM metrics between images $I_{1}$ and $I_{2}$ are given as follows:

(12) $$\begin{array}{c} PSNR\left(I_{1},I_{2}\right)=10log_{10}\left(\frac{{\left(L-1\right)}^{2}}{MSE(I_{1},I_{2})}\right)\\ MSE\left(I_{1},I_{2}\right)=\frac{1}{H\times W}\sum_{i=0}^{H}\sum_{j=0}^{W}\left|\right|I_{1}\left(i,j\right)-I_{2}\left(i,j\right){\left|\right|}_{l_{2}}, \end{array}$$

where *L* is the number of maximum possible intensity levels. *H* and *W* are the height and width of the images $I_{1}$, $I_{2}$:

(13) $$SSIM\left(I_{1},I_{2}\right)=\frac{\left(2\mu_{1}2\mu_{2}+c_{1}\right)\left(2\sigma_{12}+c_{2}\right)}{\left(\mu_{1}^{2}+\mu_{2}^{2}+c_{1}\right)\left(\sigma_{1}^{2}+\sigma_{2}^{2}+c_{2}\right)},$$

$\mu_{1}$, $\mu_{2}$ are average pixel intensities of image $I_{1}$ and $I_{2}$. $\sigma_{1}$ and $\sigma_{2}$ are the variance of image $I_{1}$ and $I_{2}$, respectively. $\sigma_{12}$ is the covariance matrix of images $I_{1}$ and $I_{2}$. The values of PSNR range from 0 to 100 and when the quality of $I_{1}$ and $I_{2}$ are the same, PSNR reaches 100. The values of SSIM range from 0 to 1 and when the quality of $I_{1}$ and $I_{2}$ are the same, PSNR reaches 1. 

These metrics are used to evaluate the enhancement performance at regions of interest defined in Figure 9. Figure 9a presents the inner lung as an RoI which is defined as a polygon formed by landmark points. Denote $R_{i}^{l}$ and $R_{i}^{r}$ as the inner left lung and the inner right lung. Figure 9b presents the outer lung which is defined by stretching the inner lung and it is implemented by applying the dilation morphological operation. Denote $R_{o}^{l}$ and $R_{o}^{r}$ as the outer left lung and the outer right lung. Figure 9c visualizes overlapping between the inner lung and the outer lung regions. In addition to the lung areas, we also consider the quality of the areas around the landmark points as given in Figure 9d. Corresponding to 10 landmark points, the areas around them are denoted as $R_{p}^{i}$, where i∈[1,..,10]. For each RoI, both the mean and standard deviation (std) of PSNR and SSIM are computed and reported in Table 2 and Table 3. Table 2 reports the mean/std of PSNR and SSIM on the lung areas, i.e., $R_{i}^{l}$, $R_{i}^{r}$, $R_{o}^{l}$, $R_{o}^{r}$, whereas Table 3 reports the mean/std of PSNR and SSIM on the areas around the landmark points, i.e., $R_{p}^{i}$. On lung areas, the PSNR values between the portable CXRs and the conventional CXRs ranges from 28.0 to 29.0 while the PSNR values between our enhanced CXRs and the conventional CXRs are improve to above 30. On the areas around landmark points, the PSNR values between the portable CXRs and the conventional CXRs ranges from 27.9 to 28.6 where as the PSNR values between our enhanced CXRs and the conventional CXRs have been increased to the range from 28.5.0 to 31.0. The high PSNR implies that the image quality from our enhanced CXRs is close to that of the conventional CXRs. Not only on PSRN, but our enhanced CXRs also obtain a higher averaging SSIM score with a lower std SSIM score compared to the potable CXRs as shown in the last two columns in Table 2 and Table 3. Generally, compared to portable CXR, the enhanced aligned portable CXR obtains a higher PSNR and SSIM, which implies that the quality of the enhanced aligned CXR is quite close to that of the conventional CXR.

**Figure 10:**
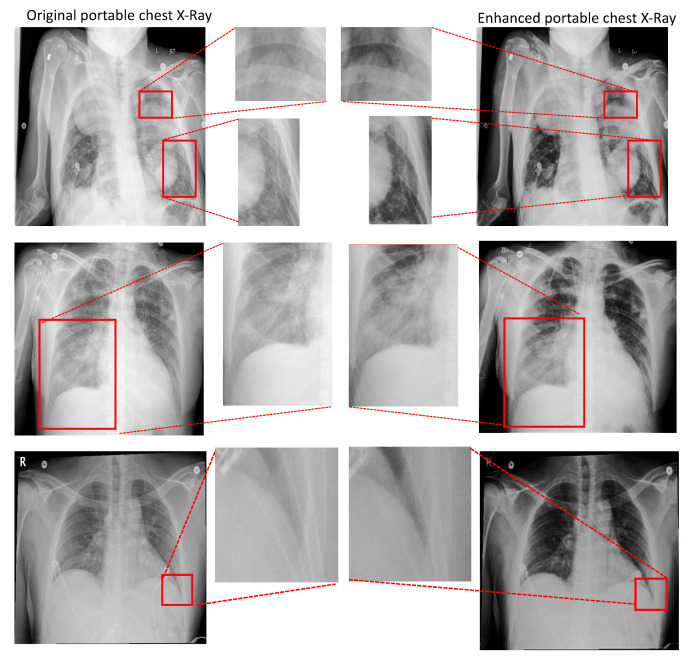
Illustrations of our radiograph enhancing results. From to top bottom—(**Top**): our enhanced CXR (right) improves contrast between the soft-tissue masses and normal lung compared to the original portable CXR (left); (**Middle**): opacity in the right lower lobe is much more conspicuous for our enhanced CXR (right) compared to the original portable CXR (left); (**Bottom**): our enhancement algorithm is able to sharpen the angle between the ribs and diaphragm. Our enhanced result is on the right, whereas the original portable CXR is on the left.

**Figure 11:**
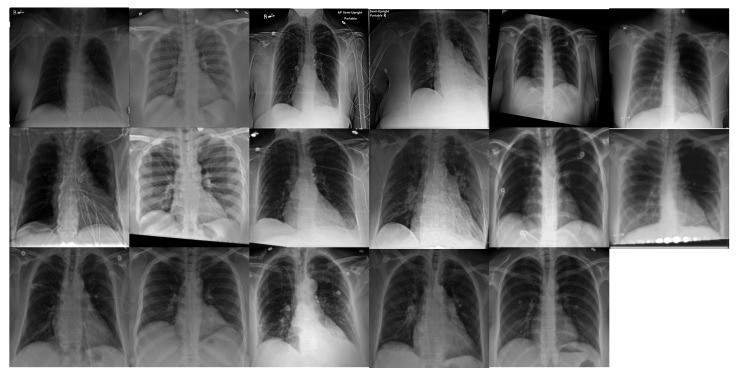
Examples of our alignment and enhancement results. From top to bottom—first row: portable CXR; second row: enhanced CXR from aligned portable CXR; third row: conventional CXR(d). Each column presents one subject. The last column is a COVID-19 case and there is no conventional CXR for this subject.

Figure 10 illustrates an enlarged view of the lung regions of original portable X-ray images and corresponding regions of our enhanced portable CXR images. It demonstrates that the proposed method produces high contrast between soft-tissue masses and normal lung compared against the original portable CXR as shown in Figure 10 (top). In addition, our enhancement network is able to provide more visible and conspicuous opacity in the lower lobe as given in Figure 10 (middle) which illustrates the right lower lobe. One of the most challenging problems of degraded portable CXR is the costophrenic angle between ribs and diaphragm. Compared to the original portable CXR, our enhanced CXR shows more sharply-pointed costophrenic angle as demonstrated in Figure 10 (bottom). Figure 11 illustrates the performance of our proposed networks where each subject is shown in one column. The portable CXRs are given in the first row whereas our enhanced CXRs are shown in the second row. The last row is the conventional CXRs that are used as groundtruth to train our proposed network.

## 5. Conclusions

In this paper, we proposed a deep learning framework to assist physicians improve their speed, treatment monitor performance, and diagnostic accuracy when using portable CXRs, which are in especially high demand in the setting of the ongoing COVID-19 pandemic. Our proposed deep neuron network consists of two components, i.e., the alignment network and PairFlow enhancement network. The experimental results have shown that our alignment network, which learns affine transformation and thin-plate spline transformation, is able to align the portable radiographs. The result is images obtained from a portable radiograph machine which are quite close to those of conventional radiographs in terms of both local topological properties and global topological properties. Our proposed PairFlow enhancement network has demonstrated its ability to enhance at least some diagnostic findings, including contrast between masses and normal lung, with better appreciation of the costophrenic angles, and improved conspicuity of opacities in the lower lobes, the latter of which is a hallmark feature of COVID-19.

## Figures and Tables

**Table 1:** MAE of CXR alignment on left lung, right lung and both lungs.

	Portable CXR vs. Conventional CXR	Aligned CXR vs. Conventional CXR
Left Lung	203.03	**42.02**
Right Lung	239.31	**38.86**
Entire CXR	221.17	**40.44**

**Table 2:** Mean/std of PSNR and SSIM for the inner left lung $R_{i}^{l}$, inner right lung $R_{i}^{r}$, outer left lung $R_{o}^{l}$, and outer right lung $R_{o}^{r}$ between the portable CXR (Por.CXR) and conventional CXR (Con.CXR) and between enhanced the CXR (Enh.CXR) and conventional CXR (Con.CXR).

RoIs	PSNR ↑		SSIM ↑	
	Por.CXR vs. Con.CXR	Enh.CXR vs. Con.CXR	Por.CXR vs. Con.CXR	Enh.CXR vs. Con.CXR
$R_{i}^{l}$	28.019/0.245	**30.273**/1.798	0.936/0.018	**0.960**/0.011
$R_{i}^{r}$	28.003/0.236	**30.437**/1.707	0.748/0.047	**0.787**/0.045
$R_{o}^{l}$	29.009/0.229	**30.474**/1.724	0.919/0.021	**0.955**/0.013
$R_{o}^{r}$	28.006/0.227	**30.522**/1.662	0.729/0.050	**0.780**/0.047
$R_{i}^{l}\cup R_{i}^{r}$	28.011/0.205	**30.407**/1.670	0.873/0.035	**0.920**/0.021
$R_{o}^{l}\cup R_{o}^{r}$	28.009/0.198	**30.498**/1.629	0.839/0.044	**0.910**/0.026

**Table 3:** Mean/std of PSNR and SSIM on the areas surrounding landmark points between the portable CXR (Por.CXR) and conventional CXR (Con.CXR) and between the enhanced CXR (Enh.CXR) and conventional CXR (Con.CXR).

RoIs	PSNR ↑		SSIM ↑	
	Por.CXR vs. Con.CXR	Enh.CXR vs. B	Por.CXR vs. Con.CXR	Enh.CXR vs. Con.CXR
P1	27.995/0.970	**28.780**/3.338	0.675/0.195	**0.723**/0.086
P2	28.079/1.126	**29.911**/2.783	0.756/0.065	**0.763**/0.062
P3	28.196/1.290	**30.025**/3.031	0.669/0.187	**0.736**/0.101
P4	27.960/0.672	**30.519**/2.453	0.706/0.076	**0.795**/0.051
P5	27.898/0.538	**29.007**/2.444	0.683/0.077	**0.736**/0.084
P6	28.159/1.097	**28.523**/3.328	0.699/0.153	**0.720**/0.098
P7	28.407/1.143	**29.576**/2.354	0.733/0.070	**0.778**/0.059
P8	28.595/1.786	**31.005**/2.201	0.776/0.063	**0.802**/0.052
P9	28.075/0.826	**30.485**/3.503	0.721/0.079	**0.737**/0.134
P10	27.940/0.676	**30.738**/2.589	0.713/0.070	**0.767**/0.067

## Data Availability

The data was provided by Department of Radiologist, University of Arkansas for Medical Sciences UAMS, Little Rock, AR 72205, USA.
